# Measuring professionals' attitudes toward persistent somatic symptoms: Development, validation, and reliability of the professionals' Attitude to Persistent Somatic Symptoms Questionnaire (PAPSS)

**DOI:** 10.1016/j.pecinn.2024.100359

**Published:** 2024-11-22

**Authors:** Denise J.C. Hanssen, Charlotte A. Spiertz, Lineke M. Tak, Judith G.M. Rosmalen

**Affiliations:** a University of Groningen, University Medical Center Groningen, Interdisciplinary Centre Psychopathology and Emotion regulation, Groningen, the Netherlands; b University of Groningen, University Medical Center Groningen, Department of Internal Medicine, Groningen, the Netherlands; c Dimence Institute for Specialized Mental Health Care, Alkura Specialist Center Persistent Somatic Symptoms, Deventer, the Netherlands

**Keywords:** Persistent somatic symptoms, Attitude, Patient-doctor communication

## Abstract

**Objective:**

The healthcare professional's attitude toward persistent somatic symptoms (PSS) seems to play an important role in access to and quality of care for patients with PSS. To encourage research on PSS attitude, we developed and validated the Professionals' Attitude to Persistent Somatic Symptoms Questionnaire (PAPSS).

**Methods:**

A list of items was developed through theory and a focus group with PSS experts, with response categories on a 5-point Likert scale ranging from “strongly disagree” to “strongly agree”. These items were then validated through a sample (*N* = 411) consisting of medical specialists, general practitioners, and psychologists. Subscales of the PAPSS were constructed using repeated factor analyses and reliability analyses.

**Results:**

Exploratory factor analyses resulted in a 15-item questionnaire with four subscales: “Perceived burden” and “Affinity” showed good reliability rates; “Perceived professional competence” and “Openness to patient-centered care” had questionable reliability rates. In general, psychologists had the most pronounced scores on subscales compared to medical specialists and general practitioners.

**Innovation:**

The PAPSS is the first questionnaire for exploring the role of the professional's attitude toward PSS; it offers opportunities for further research on the influence of attitude on treatment of PSS.

**Conclusions:**

The PAPPS is a relatively short questionnaire that can be used in both quantitative research and clinical care. However, it requires further research on psychometric qualities, including the validation of the translated versions of this questionnaire.

## Highlights

• Professionals' attitudes toward PSS play an important role in access to care.
• To measure attitude toward PSS, we developed/validated the PAPSS questionnaire*.*
• After psychometric evaluation, the PAPSS consists of 15-items and four subscales.
• Subscale scores differ between medical specialists, GPs, and psychologists.
• The PAPSS requires further research on psychometric qualities.

## 1 Introduction

Persistent somatic symptoms (PSS) - such as medically unexplained symptoms (MUS) [1], or symptoms in light of somatic symptom disorders [2] and functional disorders [3] - are associated with an impaired quality of life [[4], [5], [6], [7]], increased job loss and work problems [7], and high healthcare costs [[8], [9], [10]]. Although evidence-based treatments are available to treat PSS [11], far from all patients seem to have access to proper care.

Previous literature reviews described multiple barriers to accessing care for PSS [12,13]. One of these barriers concerns perceived difficulties between patients with PSS and professionals. Multiple studies have identified communication problems in the care for patients with PSS [14], including a “double trouble potential” with professionals and patients using different frames of reference [15] resulting in unfocused consultations [16].

This is particularly unfortunate because of indications that appropriate communication between professionals and patients with unexplained PSS may have benefits for patients, such as reduction of anxiety symptoms, finding reassurance, and increasing patient satisfaction [17]. Although clear advice is provided on the management of PSS [[18], [19], [20]], its application in clinical practice remains challenging [21].

Communication problems might be related to professionals' negative attitudes toward working with patients with PSS. In general, attitude toward both somatic (e.g. [[22], [23], [24]] and mental disorders (e.g. [[25], [26], [27]]) can have a major impact on the patient and the professional themselves. A negative attitude of the professional may affect whether or not the patient receives appropriate treatment for the illness (e.g. [24,26]). It seems that professionals' negative views on patients with PSS enable ‘passing on’ patients to another discipline, hindering receiving appropriate education about the symptoms and delaying effective treatment [28]. This cycle may in turn perpetuate the stigma surrounding having PSS [[29], [30], [31]]. In fact, one study demonstrated that most medical students already had negative attitudes toward patients with PSS, although some of them showed sympathy toward this patient group [32]. It seems as if students adopt this negative attitude from their supervisors, inferring the negative perceptions from supervisors' conversations with colleagues and students. In a study of junior doctors, this role of the supervisor's attitude was underlined, also mentioning the favorable effect of having a role model with a positive attitude toward patients with PSS [33]. Negative attitudes toward PSS thus affect patients, but may also affect the work quality and satisfaction of (future) clinicians. Indeed, the percentage of people with *persistent* somatic symptoms is relatively low, with estimates of 2.5–3 % for PSS without underlying medical cause in a general primary care population [34,35] to 16 % for symptom-based diagnoses in primary care [36]. Still, PSS make up quite a bit of the GP's workload, especially in younger doctors leading to feelings of anger and frustration when dealing with patients with PSS [37]. Indirectly, such sentiments may affect the provision of care for patients with PSS.

Unfortunately, further research on attitude in the care of PSS is limited by the absence of validated questionnaires on this topic. In the current study we describe the development, reliability and validation of the Professionals' Attitude to Persistent Somatic Symptoms Questionnaire (PAPSS). As PSS are treated at the interface of somatic and psychological care, we will validate this questionnaire in both professionals working in somatic care and professionals working in mental healthcare.

## 2 Methods

### 2.1 Development of the original items of the Professionals' attitude to persistent somatic symptoms questionnaire (PAPSS)

A multidisciplinary focus group consisting of Dutch professionals, all with extensive clinical experience in working with patients with PSS and/or substantial experience with performing scientific research in the field of PSS, formulated a long-list of items related to the professional's attitude toward PSS. For this, they used the attitude framework proposed by Breckler [38]; in this framework, it is assumed that affect, behavior and cognitions are distinct components of attitude. Likewise, the focus group formulated items on the affective (10 items), behavioral (5 items) and cognitive (7 items) level on the topic of attitude toward patients with PSS, using the input from scientific literature and experiences from clinical practice. This original list of items (Appendix A) was used to create a shorter, validated attitude scale (PAPSS) using the procedure outlined below. The professional's level of agreement for each item was measured with a 5-point Likert scale, ranging from “strongly disagree” to “strongly agree”.

### 2.2 Data collection

Two data samples were used to conduct the current validation study. The first sample was extracted from a study focusing on implementation barriers for PSS interventions (medical specialists, general practitioners, other healthcare professionals), in which the PAPSS was included. The second sample was extracted from a study focusing on attitudes toward somatic symptom disorder from a psychologist perspective. In both samples, the original list of 22 items was used to measure attitude toward PSS. The wording of the items was adjusted to the specific profession, using the term “medically unexplained symptoms” in the first sample, and using the term “somatic symptom disorder” in the second sample.

The total sample consisted of 411 participants (20.4 % medical specialists; 11.0 % general practitioners; 62.0 % psychologists, 6.6 % other healthcare professionals). Participants were recruited via either a collaborative care network for PSS (general practitioners, medical specialists, other healthcare professionals) or mental healthcare institutions (psychologists). Before responding to the survey, participants gave permission to use the anonymized data for scientific purposes. As this is an anonymous study, this study does not fall under the Medical research Involving Human Subjects Act in the Netherlands.

### 2.3 Statistical analyses

To determine the internal structure of the PAPSS, a confirmatory Maximum Likelihood factor analysis on the complete dataset with an extraction based on a pre-set number of factors (3, following the hypothesis of attitude consisting of an affective, behavioral and cognitive component) and an oblique rotation method (Promax) was performed. Goodness of fit was determined by calculating the Root Mean Square Error of Approximation (RMSEA). Next, to improve the fit and to assess the possibility of a shorter PAPSS scale, we performed a series of exploratory Maximum Likelihood factor analyses based on the eigenvalue >1 principle, using an oblique rotation method (Promax) on the 22 PAPSS items and afterwards calculating the RMSEA. Data reduction was established by repeating this procedure in case the factor solution was not satisfying, inspecting the *p*-value for the factor solution, the RMSEA and the factor structure. The solution was considered satisfying with a *p*-value > .05 or a *p*-value below 0.05 combined with an RMSEA below 0.05, and was interpreted in part from clinical meaningfulness. Whenever the *p*-value was below 0.05 and the RMSEA was higher than 0.05, the item with the lowest loading was removed from the analysis, and the factor analysis was repeated until a final factor structure was reached. Reliability of the final factor solution was determined by calculating both Crohnbach's α (as a lower limit of reliability) and Lambda 2-statistics (as an estimate of reliability).

To determine differences between professions, we performed multiple analyses of variance (ANOVAs) to compare the PAPSS scores between medical specialists, general practitioners and psychologists. Because the group with “other caregivers” (*N* = 36) involved caregivers from diverse backgrounds, we excluded this group for these analyses. Differences were considered statistically significant when *p*-values were below 0.05. All statistical analyses were performed using IBM SPSS 22.0.

## 3 Results

The total sample consisted of 411 healthcare professionals, including 255 psychologists, 75 medical specialistst, 46 general practitioners, and 35 other healthcare professionals. On average, they had 15.3 years of working experience (SD = 9.3).

### 3.1 Internal structure of the PAPSS

First, to check whether a 3-factor solution focusing on the affective, cognitive and behavioral component of attitude would be appropriate to describe the current scale, we performed factor analysis following the Maximum Likelihood Method, with a fixed number of factors (3) and oblique rotation (Promax). The results are shown in Table 1. Considering an RMSEA of 0.0561 and a *p*-value <.001, we concluded that fit of the 3-factor solution was questionable. Moreover, the overlap with the original classification was minimal (Appendix A).

**Table 1:** Factor loadings for the original PAPSS items measured in healthcare professionals (*N* = 411), assuming a 3-factor solution.

	Item	Factor 1	Factor 2	Factor 3
21.	These patients cost me a lot of energy.	**0.862**	0.040	−0.033
14.	It is difficult to deal with these patients.	**0.821**	−0.066	0.071
9.	I take more time for these patients on average.	**0.711**	0.235	0.110
13.	It is difficult to treat these patients.	**0.662**	0.146	−0.079
16.	It is difficult to communicate with these patients.	**0.658**	0.090	−0.077
17.	These patients make me feel irritated.	**0.546**	−0.238	−0.006
20.	These patients make me feel desperate.	**0.539**	−0.240	0.157
8.	I communicate the working diagnosis of persistent somatic symptoms clearly to the patient.	0.155	**0.812**	−0.255
3.	I rate my current level of knowledge about persistent somatic symptoms as adequate.	−0.031	**0.800**	−0.328
15.	When I make the working diagnosis of persistent somatic symptoms, I am certain of it.	−0.038	**0.703**	−0.486
1.	I have a particular interest in the patient population with persistent somatic symptoms.	0.121	**0.655**	0.238
10.	I can do something for these patients.	0.041	**0.600**	0.020
22.	Working with these patients gives me satisfaction.	−0.095	**0.503**	0.418
18.	I can empathize with these patients.	−0.204	**0.382**	0.099
4.	In my personal life, I know someone with persistent somatic symptoms.	0.044	**0.265**	−0.036
11.	I actively involve these patients in making decisions about the most appropriate treatment.	0.016	0.**238**	0.071
5.	A psychological explanation for persistent somatic symptoms should be considered.	0.123	**0.141**	−0.070
6.	“Persistent somatic symptoms” is a medically recognized condition.	0.019	**0.095**	−0.027
19.	I enjoy working with these patients.	−0.117	0.437	**0.521**
2.	Persistent somatic symptoms are a serious health problem.	0.123	0.236	**0.277**
7.	A biomedical explanation for persistent somatic symptoms should be considered.	−0.067	−0.104	**0.262**
12.	I usually refer these patients to a multidisciplinary team.	0.039	−0.076	**0.164**

To explore if we could achieve a better solution, Maximum Likelihood factor analyses were repeatedly performed following the eigenvalue >1 principle (Oblique, Promax rotation) for the 22 items of the PAPSS. In Table 2 we present the final solution, with an RMSEA of 0.0540 and a *p* < .001 (acceptable fit).

**Table 2:** Final factor solution for the original PAPSS items measured in healthcare professionals (*N* = 411).⁎

	Item	Factor 1	Factor 2	Factor 3	Factor 4
21.	These patients cost me a lot of energy.	**0.824**	−0.076	0.042	0.039
14.	It is difficult to deal with these patients.	**0.793**	0.004	−0.095	0.032
9.	I take more time for these patients on average.	**0.694**	0.247	0.041	−0.017
16.	It is difficult to communicate with these patients.	**0.668**	−0.032	0.106	−0.152
13.	It is difficult to treat these patients.	**0.637**	−0.034	0.103	0.006
17.	These patients make me feel irritated.	**0.520**	−0.196	−0.101	0.005
20.	These patients make me feel desperate.	**0.517**	0.004	−0.217	0.062
19.	I enjoy working with these patients.	−0.075	**0.945**	−0.132	0.014
22.	Working with these patients gives me satisfaction.	−0.069	**0.840**	−0.132	0.014
1.	I have a particular interest in the patient population with persistent somatic symptoms.	0.166	**0.721**	0.203	−0.110
2.	Persistent somatic symptoms are a serious health problem.	0.103	**0.444**	−0.084	0.172
18.	I can empathize with these patients.	−0.189	**0.359**	0.134	0.034
3.	I rate my current level of knowledge about persistent somatic symptoms as adequate.	−0.024	0.050	**0.691**	−0.055
15.	When I make the working diagnosis of persistent somatic symptoms, I am certain of it.	−0.041	−0.153	0.**650**	−0.066
8.	I communicate the working diagnosis of persistent somatic symptoms clearly to the patient.	0.133	0.104	**0.614**	0.168
11.	I actively involve these patients in making decisions about the most appropriate treatment.	−0.047	0.037	0.146	**0.716**
12.	I usually refer these patients to a multidisciplinary team.	−0.011	0.029	−0.129	**0.496**

⁎ Two items in grey (items 2 and 18) were deleted after reliability analysis, see below.

The factors were interpreted as follows. Factor 1 was named “Perceived burden” (7 items), covering items like “These patients cost me a lot of energy” and “It is difficult to communicate with these patients”. Factor 2 was named “Affinity” (3 items), including the items “I enjoy working with these patients” and “Working with these patients gives me satisfaction”. Factor 3 was named “Perceived Professional Competence”; this factor includes three items, such as “I rate my current knowledge about PSS as adequate”. The fourth factor was named “Openness to patient-centered care”, including two items on openness to patient-involvement and multidisciplinarity.

### 3.2 Reliability of the PAPSS subscales

For each subscale, a reliability analysis was performed to examine whether the deletion of items could increase reliability for that specific scale. Only for “Affinity”, the deletion of two items “Persistent somatic symptoms are a serious health problem” and “I can empathize with these patients” resulted in a higher reliability. Because additional factor analysis without these items gave no shifts in the factor structure, these items were removed from the final version of the questionnaire. The results of the final reliability analyses are presented in Table 3.

**Table 3:** Reliability coefficients for the four final subscales of the PAPSS subscales.

	Lambda 2	Cronbach's α
Perceived burden	0.859	0.854
Affinity	0.884	0.884
Perceived professional competence	0.679	0.678
Openness to patient-centered care	0.503	0.503

### 3.3 Differences between professions

To determine differences in attitudes toward PSS between the groups of professionals (medical specialists, general practitioners, psychologists), we conducted analyses of variance (ANOVAs) for each of the subscales of the PAPSS (Table 4). The professional groups significantly differed on all subscales. Scores on the “Perceived burden” subscale were highest in general practitioners and lowest in psychologists. Psychologists had the highest “Affinity” scores followed by general practitioners, with medical specialists scoring lowest on this subscale. Interestingly, medical specialists scored highest on the “Professional competence” and psychologists scored lowest, although the reported differences were small. Psychologists scored highest on “Openness to patient-centered care”, while medical specialists scored lowest on this subscale.

**Table 4:** Between-group differences and mean values (with standard deviations) of the PAPSS subscales.

	Score range	Medical specialists (*n* = 75)	General practitioners (*n* = 46)	Psychologists (*n* = 255)	F (df), *p*-value
1) Perceived burden	7–35	23.0 (4.35)	25.5 (4.3)	20.6 (4.3)	29.7 (2), *p* < .001
2) Affinity	3–15	7.9 (2.54)	8.1 (2.5)	8.8 (2.6)	4.1 (2), *p* = .018
3) Perceived professional competence	3–15	10.2 (2.02)	9.9 (1.9)	9.5 (2.2)	3.1 (2), *p* = .046
4) Openness to patient-centered care	2–10	6.4 (1.88)	7.3 (1.2)	7.7 (1.3)	21.7 (2), *p* < .001

## 4 Discussion and conclusion

### 4.1 Discussion

In the current paper, we describe the development and first steps in validation of a questionnaire for measuring attitude about PSS among healthcare professionals (PAPSS). The 15-item questionnaire consists of four subscales, i.e. 1) “Perceived burden”, 2) “Affinity”, 3) “Perceived Professional Competence”, and 4) “Openness to patient-centered care”. Subscales one and two showed good reliability. Reliability rates of the third and fourth subscales are questionable and might (partially) be explained from the low number of items. In general, scores of psychologists seem more pronounced compared to those of medical specialists and general practitioners; in fact, psychologists scored highest on subscales “Affinity” and “Openness to patient-centered care”, whereas they scored lowest on subscales “Perceived Burden” and “Perceived Professional Competence”.

Despite the major implications of attitude, few questionnaires are available to measure attitudes of professionals toward specific disorders. Corresponding with the PAPSS, the (Revised) Depression Attitude Questionnaire [39,40] consists of subscales regarding professional's confidence (matching “Perceived Professional Competence”), therapeutic optimism and pessimism (matching “Affinity” and “Perceived Burden”) and generalist perspective (matching “Openness to patient-centered care”). Given the overlap between the questionnaires' subscales, one could argue for an overall healthcare attitude questionnaire regardless of the specific condition. However, since PSS are treated at the interface of somatic and psychological care, it seemed necessary to develop an attitude questionnaire for this specific patient population, and to validate it in professionals working in both somatic and mental healthcare.

Since many different disciplines are involved in the care of PSS [41], between-profession differences regarding attitude should be studied in more depth. In the current study, it is notable that psychologists have more affinity for working with patients with PSS than both groups of physicians and they report the least burden of working with patients with PSS; psychologists also seem to be more open to patient-centered care. However, psychologists report the lowest professional competence compared to both groups of physicians, even though most evidence-based interventions for PSS are psychologically oriented [11]. Possibly, there is more uncertainty in treating PSS, mostly performed by psychologists, than in diagnosing PSS, mostly performed by medical specialists. Moreover, registered psychotherapists reported that psychological treatment of PSS is hindered by ambiguity in labeling and in the use of different exploratory models [42]. Therefore, we believe that further research on psychologists' attitudes in caring for patients with PSS, an understudied topic in the literature, is useful.

A major limitation of the current study is that we did not apply cognitive interviewing [43] to validate the items. Cognitive interviewing could have contributed to making the items better comprehensible and usable to reach conclusions about attitude toward PSS. Further, although the survey was completed anonymously, we do not rule out the possibility that social desirability influenced the results of the completed questionnaires. Also, we face possible recruitment bias, given that the different professionals were recruited through different pathways, i.e. medical specialists and general practitioners through a network for PSS care, psychologists through mental health institutions. As a result, we may have included physicians with a more positive attitude toward PSS than psychologists who are not necessarily affiliated with a network for PSS, which has possibly skewed the statistical analyses. This may also have underestimated differences in subscale scores between professionals: possibly, the differences between physicians and psychologists are more pronounced in a general population, with the expectation that physicians will have lower scores on the subscales “Affinity” and “Openness to patient-centered care”, and higher scores on “Perceived burden” compared to the scores reported in this study. Given that we used adjusted terminology in the items for somatic (“medically unexplained symptoms”) versus psychological healthcare professionals (“somatic symptom disorder”), this may also have led to a bias in results. More in general, we believe that a larger sample size and usage of additional techniques to determine the number of factors (e.g. parallel analysis) could enhance the significance of our current, preliminary results [44].

Unfortunately, the current study does not allow examining construct validity, given the lack of a gold standard. Follow-up research should examine how the current questionnaire relates to, for example, measures of professional work satisfaction or quality of care for patients with PSS. Unfortunately, because the datasets used were collected for purposes other than validating this questionnaire, no measurement instruments were included to examine convergent validity. Also, it would be helpful to examine whether the PAPSS can predict successful treatment of patients with PSS. Repeated administration of the PAPSS to determine test-retest reliability was not possible due to the unavailability of contact details of the participants in both studies. Anonymity, however, was required in our opinion, to avoid socially desirable answers. Although we included a wide range of professionals in our study, often involved professionals such as physical therapists were not included. In the future, it would be interesting to repeat this study in other relevant groups of professionals. Moreover, it is possible that the factor structure of the PAPSS is different among different disciplines, which is an interesting topic for future research. To promote comparability, however, in the current study we chose to analyze the data at the level of the complete group of healthcare providers.

### 4.2 Innovation

The PAPSS is a short questionnaire that can easily be included in research on the involvement of healthcare providers in the care of patients with PSS. Recentely, translated versions of this questionnaire were added to a large-scale European survey comparing the care for PSS in different European countries [45]. This future multi-language database will allow the factor structure of this translated questionnaire to be determined, and compared with the structure of the Dutch-language version. For the Dutch version, the present study discusses the first steps in the development of the PAPSS. A clear and easily interpretable factor structure emerged after statistical analyses. Since patients with PSS are treated partly in somatic and partly in psychological care, we believe it is strong that we involved both medical (general practitioners, medical specialists) and psychological healthcare providers (psychologists) in this study. That being said, in the current study, it was not possible to collect data focusing on content validity, both divergent and divergent validity. Determining content validity is a high priority, and should be the focus for future research on the validation of the PAPSS. The recently developed scale for measuring stigma in PSS and functional syndromes can be helpful in this regard (the Persistent Somatic Symptom Stigma Scale for Healthcare Professionals, PSSS-HCP, [46,47]). Indeed, this newly developed PSSS-HCP is a related questionnaire, but with the main focus on societal judgment (i.e. stereotypes, prejudices, discrimination), while attitudes mostly reflect personal beliefs or feelings.

### 4.3 Conclusion

Although the questionnaire will require further research on psychometric qualities, most importantly regarding content validity, the PAPSS offers opportunities for further research on the influence of attitude on treatment of PSS. Future research may additionally focus on application of the PAPSS in clinical practice, for awareness of attitude and opportunities to change this attitude for the benefit of the patient ánd the professional [48].

## Funding

This research did not receive any specific grant from funding agencies in the public, commercial, or not-for-profit sectors.

## CRediT authorship contribution statement

**Denise J.C. Hanssen:** Writing – review & editing, Writing – original draft, Visualization, Project administration, Methodology, Investigation, Formal analysis, Data curation, Conceptualization. **Charlotte A. Spiertz:** Writing – review & editing, Project administration, Data curation. **Lineke M. Tak:** Writing – review & editing, Project administration, Data curation. **Judith G.M. Rosmalen:** Writing – review & editing, Supervision, Resources, Formal analysis, Data curation.

## Declaration of competing interest

The authors declare that they have no known competing financial interests or personal relationships that could have appeared to influence the work reported in this paper.

## Figures and Tables

**Table:** 

Attitude component	Item
Cognitions	1. I have a particular interest in the patient population with persistent somatic symptoms.
	2. Persistent somatic symptoms are a serious health problem.
	3. I rate my current level of knowledge about persistent somatic symptoms as adequate.
	4. In my personal life, I know someone with persistent somatic symptoms.
	5. A psychological explanation for persistent somatic symptoms should be considered.
	6. “Persistent somatic symptoms” is a medically recognized condition.
	7. A biomedical explanation for persistent somatic symptoms should be considered.
Behavior	8. I communicate the working diagnosis of persistent somatic symptoms clearly to the patient.
	9. I take more time for these patients on average.
	10. I can do something for these patients.
	11. I actively involve these patients in making decisions about the most appropriate treatment.
	12. I usually refer these patients to a multidisciplinary team.
Affect	13. It is difficult to treat these patients.
	14. It is difficult to deal with these patients.
	15. When I make the working diagnosis of persistent somatic symptoms, I am certain of it.
	16. It is difficult to communicate with these patients.
	17. These patients make me feel irritated.
	18. I can empathize with these patients.
	19. I enjoy working with these patients.
	20. These patients make me feel desperate.
	21. These patients cost me a lot of energy.
	22. Working with these patients gives me satisfaction.

**Table:** 

Attitude component	Item
Cognitions	1. Ik heb bijzondere interesse in de patiëntenpopulatie met aanhoudende lichamelijke klachten.
	2. Aanhoudende lichamelijke klachten zijn een serieus gezondheidsprobleem.
	3. Mijn huidige kennisniveau over aanhoudende lichamelijke klachten beoordeel ik als voldoende.
	4. In mijn persoonlijk leven ken ik iemand met aanhoudende lichamelijke klachten.
	5. Er is een psychologische verklaring voor aanhoudende lichamelijke klachten te vinden.
	6. “Aanhoudende lichamelijke klachten” is een medisch erkende aandoening.
	7. Er is een biomedische verklaring voor aanhoudende lichamelijke klachten te vinden.
Behavior	8. Ik communiceer de werkdiagnose aanhoudende lichamelijke klachten helder naar de patiënt.
	9. Ik neem gemiddeld meer tijd voor deze patiënten.
	10. Ik kan iets betekenen voor deze patiënten.
	11. Ik betrek deze patiënten actief bij de besluitvorming over de meest geschikte behandeling.
	12. Ik verwijs deze patiënten meestal naar een multidisciplinair team.
Affect	13. Het is moeilijk om deze patiënten te behandelen.
	14. De omgang met deze patiënten ervaar ik als lastig.
	15. Wanneer ik de werkdiagnose aanhoudende lichamelijke klachten stel ben ik daar zeker van.
	16. Met deze patiënten is moeilijk te communiceren.
	17. Door deze patiënten voel ik mij geïrriteerd.
	18. In deze patiënten kan ik mij goed inleven.
	19. Met deze patiënten werk ik graag.
	20. Door deze patiënten voel ik mij wanhopig.
	21. Deze patiënten kosten mij veel energie.
	22. Het werken met deze patiënten geeft mij voldoening.

**Table:** 

Subscale	Item
Perceived burden	1. These patients cost me a lot of energy. 2. It is difficult to deal with these patients. 3. I take more time for these patients on average. 4. It is difficult to communicate with these patients. 5. It is difficult to treat these patients. 6. These patients make me feel irritated. 7. These patients make me feel desperate.
Affinity	8. I enjoy working with these patients. 9. Working with these patients gives me satisfaction. 10. I have a particular interest in the patient population with persistent somatic symptoms.
Perceived professional competence	11. I rate my current level of knowledge about persistent somatic symptoms as adequate. 12. When I make the working diagnosis of persistent somatic symptoms, I am certain of it. 13. I communicate the working diagnosis of persistent somatic symptoms clearly to the patient.
Openness to patient-centered care	14. I actively involve these patients in making decisions about the most appropriate treatment. 15. I usually refer these patients to a multidisciplinary team.
